# Physical inactivity and perceived environmental factors: a cross-sectional study among civil servants in Abia State, Southeastern Nigeria

**DOI:** 10.11604/pamj.2022.42.74.31878

**Published:** 2022-05-26

**Authors:** Chidinma Ihuoma Amuzie, IkeOluwapo Ajayi, Eniola Bamgboye, Chukwuma David Umeokonkwo, Christian Obasi Akpa, Ugonma Okpechi Agbo, Uche Ngozi Nwamoh, Michael Izuka, Muhammad Shakir Balogun

**Affiliations:** 1 Nigeria Field Epidemiology Training Program, Abuja, Nigeria,; 2 Department of Community Medicine, Federal Medical Centre, Umuahia, Abia State, Nigeria,; 3 Department of Epidemiology and Medical Statistics, Faculty of Public Health, College of Medicine, University of Ibadan, Ibadan, Nigeria,; 4 Department of Community Medicine, Alex Ekwueme Federal University Teaching Hospital, Abakaliki, Ebonyi State, Nigeria,; 5 Abia State Ministry of Health, Umuahia, Abia State, Nigeria

**Keywords:** Built environment, exercise, Nigeria, neighbourhood, perception, prevalence, residence characteristics, sedentary behaviour

## Abstract

**Introduction:**

physical inactivity has been identified as the fourth leading risk factor for global mortality due to non-communicable diseases. Prevalence rates of 91.0% and 62.2%, have been documented among civil servants in the northern and southern parts of Nigeria, respectively. There is a paucity of data regarding the relationship between physical inactivity and environmental factors among civil servants in the State. This study assessed the prevalence and perceived environmental factors associated with physical inactivity among civil servants in Abia State Nigeria.

**Methods:**

we conducted a cross-sectional study in which we recruited 440 civil servants using a multistage sampling technique. We used an interviewer-administered structured questionnaire to collect data on sociodemographic, physical activity, and neighbourhood environmental attributes. Descriptive, bivariate and multivariate analysis were done. The level of significance was set at 5%.

**Results:**

the mean age of the respondents was 39.0±9.2 years, and 61% were females. The prevalence of physical inactivity was 48.4% (95%CI: 43.7%-53.2%). The putative environmental factors included perceptions of low residential density areas, perceived absence of neighbourhood sidewalks, perceived unavailability of bicycling facilities and the perception of an unsafe neighbourhood due to night crimes. The predictor of physical inactivity was the perceived absence of neighbourhood sidewalks (aOR=2.02, 95%CI: 1.10-3.73).

**Conclusion:**

in this study, physical inactivity is prevalent among civil servants in Abia State. The need for the stakeholders in collaboration with the Ministry of Environment to focus on the provision of sidewalk facilities, layout of residential areas and limit security risks in the State to enhance physical activity is highlighted.

## Introduction

Physical inactivity has been identified as one of the leading risk factors for mortality globally [1]. The burden of physical inactivity is increasing in low- and middle-income countries, with a major concern about the simultaneous rise in the prevalence of Non-communicable Diseases (NCDs) and their global public health threats [2]. Physical inactivity contributes to an attributable risk of 6% of global deaths due to NCDs and 9% of premature mortality [3,4]. In 2018, World Health Organization (WHO) reported that more than one in four adults globally (28%, or 1.4 billion people), were physically inactive [5]. These NCDs impose a large burden on the health system already drained by the rising prevalence of infectious diseases in low-income countries and lead to a fall in productivity following absenteeism. In Nigeria, there has been a consistent rise in the prevalence of physical inactivity among civil servants [6-8]. In Abia State, studies have reported a concomitant rise in the burden of NCDs and the associated risk factors among the population [9,10]. Assessing the determinants of physical inactivity among civil servants whose population constitutes the greater percentage of the workforce, will help to address the burden associated with physical inactivity of a larger proportion of the population involved in the economic growth of the State [11,12]. One of the key interventions of the national policy and strategic plan on NCDs focuses on the provision of supportive and environmentally safe active commuting [13]. The built environment plays a contributory role in determining the level of Physical Activity (PA). People's physical activity patterns are largely dependent on how they perceive and interpret their neighbourhood [14]. In line with the national policy and strategic plan on NCDs, there is a need to provide data for the monitoring of risk factors for NCDs which includes physical inactivity as well as research hypotheses on NCD prevention and control among civil servants [13]. It is important to generate more data that will augment the objective environmental assessment among this subgroup of the population to aid surveillance activities among them [15]. We assessed the prevalence and perceived environmental factors associated with physical inactivity among civil servants in Abia State.

## Methods

**Study design and setting:** a descriptive cross-sectional study was conducted among the civil servants in Umuahia from January - March 2019. Umuahia is the capital city of Abia State in southeastern Nigeria. The city had a projected population of 303,787 in 2018 [16]. The Abia State Secretariat is the State Government's administrative Headquarters, located in Umuahia. The total staff strength of the ministries in Abia State, according to the Civil Service Commission, was approximately 5000 as of 2016. The State Secretariat accommodates the government ministries, departments and agencies. There are no recreational facilities such as a staff sports club within the Secretariat.

**Study population:** they included civil servants in the State. A civil servant is an employee in any federal, state, or local government establishment, excluding the military and police. Participants who were permanent residents in Umuahia were included in the study. Pregnant civil servants, contract/ad-hoc staff, and respondents who had comorbidities that hindered physical activities such as arthritis were excluded. A total of 440 participants were recruited using the multistage sampling technique. Stage one: eight of the twenty-seven ministries in the State were selected using the balloting technique. These included the ministries of justice, works, health, education, information/strategy, agriculture, lands/survey and urban planning. Stage two: in each of the eight ministries selected, fifty-five respondents were selected. Respondents were stratified into males and females and proportionate to size sampling was done. They were recruited consecutively from the offices until the allocated sample for each stratum was reached.

**Measurement of variables:** the dependent variable was physical inactivity. The independent variables included socio-demographic factors such as age, sex, marital status, place of residence, educational status, religion, denomination, average monthly income, and job cadre; and perceived environmental factors including residential area density, street connectivity, aesthetics, neighbourhood safety, social environment, access to termini (bus stops), sidewalks, and bicycling facilities. Physical activity was categorized into three levels: low (inactive), moderately active, and highly active, as defined by the IPAQ criteria. Category 1: low (inactive), these were respondents who reported no activity or whose activity was not enough to meet categories 2 or 3. Category 2: (moderate), respondents in this category met any one of the following three (3) criteria: three or more days of vigorous activity of at least 20 minutes per day; five or more days of moderate-intensity activity or walking of at least 30 minutes per day; OR five or more days of any combination of walking, moderate-intensity, vigorous-intensity activities achieving a minimum of at least 600 MET-min/week. Category 3: (high), respondents in this category met any of the following two (2) criteria: At least three days of vigorous-intensity activity with a total of 1500 MET-minutes a week OR seven or more days of any combination of walking, moderate-intensity or vigorous-intensity activities achieving a minimum of at least 3000 MET-minutes/week. These three groups were then re-categorized as sufficiently physically active (categories 2 and 3) or physically inactive (category 1). To determine MET, frequency, duration, and intensity for each type (vigorous, moderate, and walking) of PA was utilized. One MET is defined as the energy expenditure while sitting quietly for one hour, which for the average adult is approximately 3.5 ml of oxygen/kg body weight/min with MET values obtained from the compendium of physical activities by Ainsworth 1993 [17]. A “neighbourhood” was defined as the area within a 10-to 15-minute walk from home [18].

**Data collection tool and methods:** a pre-tested interviewer-administered semi-structured questionnaire was used to collect information from the participants. Physical activity and the environmental variables were measured using standard questionnaires: the International Physical Activity Questionnaires short form (IPAQ-SF) and the Physical Activity Neighbourhood Scale (PANES) [19,20]. The IPAQ-SF designed by WHO, has been validated and is widely used to assess physical activity patterns. The test-retest reliability (ICC= 0.33-0.73) and concurrent validity (p=0.78-0.92) of the IPAQ-short form in Nigeria were found to be good [21]. The PANES is a short-form environmental measure that uses single items instead of multi-item scales. The reliability of this questionnaire has been established in Nigeria with a reliability coefficient of 0.60 [22]. Further adaptation to the Nigerian context has been done and the test-retest reliability (ICC=0.62-1.00) was good. The questionnaire was pre-tested to detect and correct possible challenges before the initiation of the study by using fifty (approximately 10% of the sample size) civil servants in the Ministry of Finance. This ministry was not selected for the study.

**Sample size determination:** the sample size for this study was calculated using the sample size formula for single-proportion studies. The formula is given as


$$N=\frac{Z\alpha^{2}pq}{d^{2}}$$

with 'p' (prevalence of physical inactivity) given at 62.2% from a previous study among civil servants in Ibadan, Oyo State [6], with a 5.0% level of significance and a 5.0% error margin. The minimum sample size was adjusted to accommodate a 10% non-response rate. The calculated sample size after adjusting for non-response was 440.

**Statistical analysis:** data coding, entry, cleaning, and editing for inconsistencies were done using the IPAQ and PANES guidelines. Analysis was done using Microsoft Excel and Epi Info 7.2. Mean and standard deviation were calculated for continuous variables such as age and number of living children, while the median and interquartile range (IQR) were computed for continuous variables not normally distributed, such as average monthly income. The prevalence of physical inactivity was estimated. The association between the prevalence of physical inactivity and environmental factors was examined using the Χ^2^-test. For the “income” variable, two missing responses were noted and the remaining proportion was used for analysis. Logistic regression analysis was done to identify the independent predictors of physical inactivity at a 5% level of significance.

**Ethical consideration:** ethical approval was obtained from the Abia State Ministry of Health Ethical Review Board with reference number (AB/MH/AD/904/T.163). Written informed consent was obtained from all respondents before administering the questionnaire. The confidentiality of the respondents was maintained by removing their personal information.

## Results

**Sociodemographic characteristics of respondents:** four hundred and forty (440) respondents participated in this study. The mean age of the study respondents was 39.0±9.2 years. Males had a slightly higher mean age (39.2±9.2 years) compared to females (38.8±9.1 years). Two hundred and ninety-two (66.4%) of respondents are between the ages of 25 and 44. There were more females 270 (61.4%) than males. Three hundred and three (68.9%) resided in urban locations, with the majority (65.9%) of them having a minimum of tertiary education. About 439 (99.8%) were Christians, with more than 40% belonging to the Pentecostal denomination. Only one respondent was a Muslim. Among the civil servants studied, 239 (54.3%) were married. The majority of the respondents, 315 (71.9%), were from the senior cadre and 199 (45.4%) of them were in the ₦36,000 - ₦71,999 monthly income categories. The median monthly income was 40,000 Naira (range: ₦18,000 - ₦380,000). One hundred and ninety (43.2%) of them had no functional vehicle in their household. For the variable (Income), we had two missing responses (n=438) (Table 1).

**Table 1:** sociodemographic characteristics of civil servants in Umuahia, Abia State, 2019 (N=440)

Sociodemographic variables	Frequency (n)	Percentage (%)
Age group (years)		
<25	28	6.4
25-44	292	66.4
45-64	120	27.3
Sex		
Male	170	38.7
Female	270	61.4
Place of residence		
Rural	137	31.1
Urban	303	68.9
Educational status		
Primary	20	4.6
Secondary	130	29.6
Tertiary	290	65.9
Denomination		
Catholic	95	21.6
Orthodox	154	35.1
Pentecostal	190	43.3
Cadre*		
Junior (GL 1-6)	123	28.1
Senior (≥GL 7)	315	71.9
Marital Status		
Married	239	54.3
Single	187	42.5
Separated (divorced/widowed)	14	3.2
Monthly income (Naira ₦) *		
18,000-35,999	168	38.4
>36,000-71,999	199	45.4
72,000-89,999	13	3.0
≥90,000	58	13.2
Number of functional vehicles owned ┼		
0	190	43.2
1	145	33.0
2	86	19.6
≥3	19	4.3

* n= 438 ^┼^ cars, trucks motorcycles

**Figure 1:**
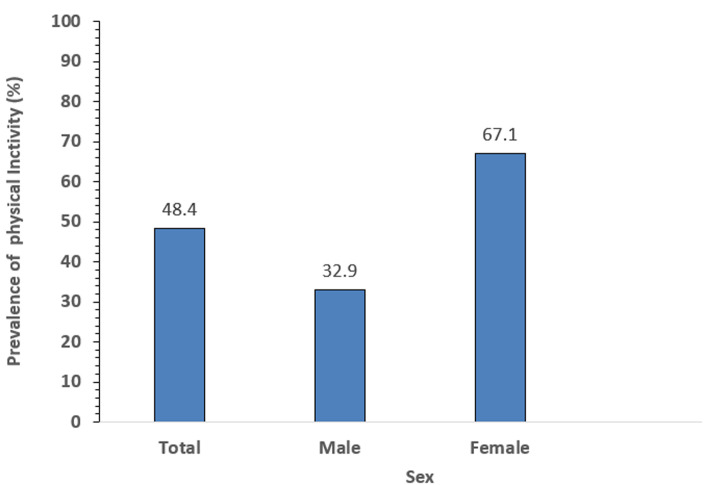
prevalence of physical inactivity by sex among the respondents (N=440)

**Prevalence of physical inactivity:** two hundred and thirteen of them (48.4%) were physically inactive (95% CI: 43.7% - 53.2%) and this was similar in the urban 145 (47.9%) and rural 68 (49.6%) areas. The females had a higher prevalence of physical inactivity of 143 (67.1%) compared to the males 70 (32.9%) (Figure 1).

**Distribution of perceived environmental factors by respondents:** the majority of the respondents 370 (84.1%) perceived shops to be within walking distance of their homes. The majority of 341 (77.5%) of the respondents thought it was ten to fifteen minutes away from work to bus stops. Only 167 (38.0%) reported the presence of sidewalks in the neighbourhood. Ninety-eight (22.3%) had a positive perception of the presence of low-cost recreational facilities in the neighbourhood. Two hundred and forty-four (55.5%) thought that the neighbourhood was unsafe due to the high night crime rate. Conversely, few of the participants 90 (20.5%) perceived insecurity in the neighbourhood due to the high daytime crime rate. Three hundred and seventeen (72.0%) noticed seeing physically active people in the neighbourhood. However, only 75(17.0%) had a positive perception of well-maintained, unobstructed sidewalks and bicycle facilities in their neighbourhood. A greater proportion of 370 (84.1%) of the respondents perceived the presence of easy walking distances in their neighbourhood (Table 2).

**Table 2:** perceived neighbourhood environmental characteristics among civil servants in Umuahia, Abia State 2019, (N=440)

Variables	Agree (%)	Disagree (%)	Not applicable (%)	Unsure (%)
High residential density	340 (77.3)	100 (22.7)	-	-
Shops within walking distance around my home	370 (84.1)	52 (11.8)	5 (1.1)	13 (3.0)
10 to 15 minutes-walk to a transit/bus stop from my home	341 (77.5)	84 (19.1)	6 (1.4)	9 (2.0)
Presence of sidewalks in my neighborhood	167 (38.0)	118 (26.8)	127 (28.9)	28 (6.4)
Presence of bicycling facilities in or near my neighborhood	75 (17.0)	191 (43.4)	137 (31.1)	37 (8.4)
Presence of free/low-cost recreational facilities	98 (22.3)	208 (47.3)	100 (22.7)	34 (7.7)
Unsafe neighborhood due to night crime	244(55.5)	160 (36.4)	17 (3.9)	19 (4.3)
Unsafe neighborhood due to day crime	90 (20.5)	318 (72.3)	15 (3.4)	17 (3.9)
Many people are physically active in my neighborhood	317 (72.0)	90 (20.5)	17 (3.9)	16 (3.6)
Traffic unsafe for bicycling	119 (27.0)	269 (61.1)	32 (7.3)	20(4.5)
Traffic unsafe for walking	109 (24.8)	294 (66.8)	20 (4.5)	17 (3.9)
Interesting things to see in my neighborhood	288(65.5)	122 (27.7)	14 (3.2)	16 (3.6)
Well-maintained and unobstructed sidewalks	84 (19.1)	178 (40.5)	135 (30.7)	43 (9.8)
Well-maintained and unobstructed bicycle facilities	75 (17.0)	191 (43.4)	137 (31.1)	37 (8.4)
Many four-way intersections in my neighborhood	169 (38.4)	116 (26.4)	103 (23.4)	52 (11.8)
Places within easy walking distance in my neighborhood	370 (84.1)	54 (12.3)	2 (0.5)	14 (3.2)

**Perceived environmental factors associated with physical inactivity:** only four out of the 16 environmental variables were significantly associated with physical inactivity. Respondents who perceived their neighbourhood to have low residential area density were 42% less likely to be physically inactive than their counterparts (COR=0.58; 95% CI: 0.37-0.91). Respondents who perceived their neighbourhood as not having sidewalks were more likely to be inactive (COR=2.58; 95%CI: 1.59-4.20). Those who perceived an unsafe neighbourhood due to crime at night were 68% more likely to be inactive (COR=1.68; 95%CI: 1.12-2.51). Similarly, those who perceived their neighbourhood as not having bicycling facilities, paths, or lanes in it were 74% more likely to be physically inactive. (COR=1.74; 95%CI: 1.06-2.87). In multivariate analysis, only the perceived unavailability of sidewalks was found to be significant. (aOR=2.02; 95%CI: 1.10-3.73) (Table 3).

**Table 3:** perceived neighbourhood environmental factors associated with physical inactivity among civil servants in Umuahia, Abia State, 2019

Variables	Physical activity		cOR (95% CI)	P-value	AOR (95% CI)	P-value
	Inactive (n%)	Active (n%)				
Residential density						
Low	38 (38.0)	62 (62.0)	0.58(0.37-0.91)	0.018*	0.78(0.39-1.41)	0.372
High	175 (51.5)	165 (48.5)	1		1	
Shops within walking distance (n=422)						
Disagree	20 (38.5)	32 (61.5)	0.63(0.34-1.13)	0.119	-	-
Agree	185 (50.0)	185 (50.0)	1		-	
Transits/bus stops (n=425)						
Disagree	40(47.6)	44 (52.3)	0.99(0.62-1.60)	0.976	-	-
Agree	163 (47.8)	178 (52.2)	1		-	
Availability of neighborhood sidewalks (n=385)						
Disagree	66(55.9)	52 (44.1)	2.58 (1.59-4.20)	<0.001*	2.02 (1.10-3.73)	0.023
Agree	55(32.9)	112 (67.1)	1		1	
Availability of bicycle facilities (n=277)						
Disagree	78 (47.3)	87 (52.7)	1.74 (1.06-2.87)	0.027*	1.01 (0.53-1.91)	0.975
Agree	38 (33.9)	74 (66.1)	1		1	
Availability of recreational facilities (n=306)						
Disagree	92 (44.2)	116 (55.8)	1.49 (0.91-2.46)	0.114	-	-
Agree	34 (34.7)	64 (65.3)	1		-	
Night crime (n=404)						
Agree	132 (54.1)	112 (45.9)	1.68 (1.12-2.51)	0.012*	1.37 (0.78-2.36)	0.251
Disagree	66 (41.3)	94(58.7)	1		1	
Day crime (n=408)						
disagree	163 (51.3)	155 (48.7)	1.51 (0.94-2.42)	0.089	-	-
Agree	37(41.1)	53(58.9)	1		-	
Seeing people active (n=407)						
Disagree	42 (46.7)	48 (53.3)	0.89 (0.56-1.42)	0.632	-	-
Agree	157 (49.5)	160 (50.5)	1		-	
Traffic against bicycles (n=388)						
Disagree	131(48.7)	138 (51.3)	0.97 (0.63-1.49)	0.872	-	-
Agree	59 (49.6)	60(50.4)	1			
Traffic against walk (n=403)						
Disagree	152 (51.7)	142 (48.3)	1.52 (0.98-2.37)	0.063		
Agree	45 (41.3)	64 (58.7)	1			
Interesting things in the neighbourhood (n=410)						
Disagree	65 (53.3)	57 (46.7)	1.26 (0.82-1.92)	0.290	-	-
Agree	137 (47.6)	151 (52.4)				
Well-maintained sidewalks(n=262)						
Disagree	77 (74.0)	101 (63.9)	1.61 (0.93-2.77)	0.086	-	-
Agree	27 (26.0)	57 (36.9)	1		-	
Well-maintained bicycle facilities (n=266)						
Disagree	81 (42.4)	110 (57.6)	1.66 (0.94-2.94)	0.077	-	-
Agree	23 (30.7)	52 (69.3)	1		-	
Four-way intersections (n= 285)						
Disagree	48 (41.4)	68 (58.6)	0.99 (0.62-1.61)	0.995	-	-
Agree	70 (41.4)	99 (58.6)	1		-	
Places within walking distance (n=424)						
Disagree	25 (53.7)	29 (46.3)	0.90 (0.51-1.60)	0.719	-	-
Agree	181 (48.9)	189 (51.1)	1			

COR: Crude Odds Ratio; aOR: Adjusted Odds Ratio, CI: Confidence Interval

## Discussion

This study aimed to determine the prevalence of physical inactivity and the associated perceived environmental factors. In this study, the prevalence of physical inactivity was moderately high among the respondents and there were some significant perceived environmental correlates. The findings of this study showed that nearly half of the respondents were physically inactive. A similar finding was observed in a study which reported 43.3% inactivity among senior civil servants in Lagos State [23]. Additionally, this result is in keeping with a systematic review which documented a range of 25-57% in studies assessing the prevalence of physical inactivity in Nigeria [24]. The prevalence of physical inactivity in this study is also similar to that found in many African countries surveyed in the global inactivity study [25]. However, this result contrasts the findings in Kaduna and Oyo States, which reported a prevalence of 91% and 67%, respectively, among civil servants [6,7]. The higher prevalence in these studies can be explained in part due to the fact that only leisure-time-related physical activity was used to assess physical inactivity among the respondents in these States. Health education interventions and sensitization programs among civil servants on the effects of physical inactivity should be advocated. This will contribute to efforts to achieve a global target of reducing physical inactivity by 10% in the year 2025 [26]. Perceived unavailability of sidewalks in the neighbourhood was a significant predictor of physical inactivity in this study. This was not an unexpected finding, as it is similar to the findings of a study conducted among adults in South Africa [27]. Furthermore, a report by a group of researchers was in consonance, as they reported sidewalks and bicycling facilities as having strong positive associations with physical activity across the 11 countries in their study [18]. However, this finding contrasts with the results of a quasi-experimental study conducted in Houston, USA among adults, which reported that improving or building sidewalks was not an indication of an enhanced level of physical activity among those who were inactive [28]. Brisk walking is a form of physical activity common to the population. Most of the time, people are hindered from engaging in it due to poorly defined walk lanes or obstruction from other road users. The State Government should work with the Ministry of Environment to construct or renovate neighbourhood infrastructures. This will encourage active participation in brisk walking and other walk-related forms of exercise.

Respondents in this study who perceived their neighbourhood as a low-density residential area had lower odds of physical inactivity compared to those who perceived it as a high-density residential area. However, this was not significant in the multivariate analysis. This is consistent with WHO fact sheets which documented that high-density traffic and urbanization, as seen in high-density residential areas, augment physical inactivity [26]. This is inconsistent with a study from eleven countries in five continents that studied the variations of perceived neighbourhood attributes and physical activity, where there was a positive association with high residential areas in most countries [29]. A possible explanation for this could be that those who perceive low residential area density are likely to have open areas around their neighbourhood that will encourage easy access to various places, as opposed to high-density areas that are prone to access road obstructions. In Abia State, people who engage in outdoor physical activities prefer areas with less obstruction and open space. Policies on physical inactivity management should target addressing a comprehensive strategy for town planning. Another significant correlate was a perceived unsafe neighbourhood due to night crimes. However, in the present study, it was not a predictor for physical inactivity. This is consistent with studies in Nigeria, where higher levels of perceived safety augment physical activity [30,31].

A comparable finding was also noted in a study conducted in Missouri, USA [22]. Additionally, a meta-analysis conducted in the USA by a group of researchers observed higher odds of physical inactivity in areas with a higher level of crime [32]. In Abia State, the security status is grossly suboptimal. People who perceive their neighbourhood as being safe are prone to attain the recommendation for physical exercise activities [33]. There are known lapses in the security system of the country. This makes people feel insecure and eventually hibernate in their homes, preventing them from engaging in physical activity. Security agencies should prioritize safety measures in the neighbourhood. Environmental safety measures should be enforced by the security agencies, to maintain low level of crimes in the neighbourhood. At the community level, we recommend the use of vigilante groups to secure the neighbourhoods. The major strength of this study is that it provides insight into the pattern of physical inactivity among civil servants in Abia State, in line with the national policy and strategic plan on NCDs, for future studies. Additionally, the study generates more information, given the sparse literature on environmental factors among the study population. Nevertheless, the limitations of this study included a possible recall bias, which was mitigated by maintaining a short interval recall time (7 days). Secondly, the use of self-reported measures for physical activity is likely to introduce social-desirability bias. This was mitigated by ensuring that responses were well evaluated before they were recorded. Additionally, the outcome variable was not objectively measured with devices (pedometers) which could have led to under-reporting of physical inactivity. To increase the generalizability of the findings, the sample was selected to be representative of the study participants.

## Conclusion

The prevalence of physical inactivity was moderately high among the civil servants in Abia State with significant perceived environmental factors. Residential density, unsafe neighbourhood due to night crimes, neighbourhood sidewalks, and bicycling facilities were the significant perceived environmental factors. The predictor of physical inactivity was the perceived absence of neighbourhood sidewalks. Health education interventions on the reduction of physical inactivity should be conducted at workplaces. These findings suggest that public health interventions should target environmental factors in the context of rising NCDs.

### What is known about this topic

- The prevalence of physical inactivity has been documented among civil servants in different regions of the globe;
- Perceptions of the built environment are related to physical activity.

### What this study adds

- This study provides a physical inactivity measure among civil servants in Abia State that will inform policy strategies geared towards the prevention and control of physical inactivity;
- It gives an insight into the associated environmental correlates within the context of the study area.
